# Food Adulteration and Consumer Awareness in Dhaka City, 1995-2011

**Published:** 2014-09

**Authors:** Sharifa Nasreen, Tahmeed Ahmed

**Affiliations:** ^1^James P. Grant School of Public Health, BRAC University, Dhaka 1212, Bangladesh; icddr,b, GPO Box 128, Dhaka 1000, Bangladesh

**Keywords:** Adulterants, Consumer awareness, Food adulteration, Food hazard, Food safety, Temporal trend, Bangladesh

## Abstract

We conducted this study to investigate the magnitude of food adulteration during 1995–2011 and consumer awareness in Dhaka city. We reviewed results of food sample testing by Public Health Food Laboratory of Dhaka City Corporation, Bangladesh Standards and Testing Institution, Consumers Association of Bangladesh publications, reports from lay press, including those on mobile magistrate court operations. We conducted a cross-sectional survey among 96 residents of Dhaka city, using a structured questionnaire in 2006. The overall proportion of food samples adulterated decreased during 2001-2005, and 40-54% of daily-consumed food was adulterated during 1995-2011. More than 35 food items were commonly adulterated. Consumers considered expiry date and quality or freshness as the best criteria while buying packaged and open food items respectively; only 11 (12%) respondents considered approval of regulatory authority for buying packaged food items. More than half of the food consumed in Dhaka city is adulterated, which warrants actions by the Government, the industry, and the consumers.

## INTRODUCTION

Food safety, an important global public health issue to ensure sound health, refers to addressing “all those hazards, whether chronic or acute, that may make food injurious to the health of the consumer” (1). Important food hazards include microbial hazards, pesticide residues, misuse of additives, chemical contaminants, including biological toxins and adulteration. Although microbiological contamination and chemical hazards have received most attention, it is recognized that food adulteration and food fraud should not be neglected considering their role in public health (2). Food adulteration includes various forms of practices, such as mixing, substituting, concealing the quality of food by mislabelling, putting up decomposed or expired food, and adding toxic substances (3). It is an age-old problem that affects people at all societal strata. The consequences of food adulteration are two-fold for the consumers: the economic loss by paying more for lower-quality food items and the health hazards. The health hazards can result from either addition of deleterious substances or removal of a vital component (4). Some adulterants may even lead to death (1,3).

Most of the food items collected from the respondents’ residents were found adulterated in a study conducted in Haryana, India, and the main adulterants in food samples included water in milk, chalk powder in turmeric powder or sugar, artificial colour in chili powder, water-soluble colour in green and black gram, artificial colour in chickpea flour, and essential oil removed from cardamom (5). More than half of the food samples tested during 2002 at the Institute of Public Health in Dhaka were adulterated; among the samples tested, 100% samples of butter oil and *banaspati dalda*, 90% condensed milk/sweetmeats, 72.3% *ghee* and honey, and 57.2% cow's milk were adulterated (6). During 2002-2003, Bangladesh Standards and Testing Institute (BSTI) had 250 surveillance team/mobile courts that collected 226 food samples from open market for testing, issued 117 show-cause notices to manufacturers for substandard products, cancelled 45 trade licenses, and undertook 35 legal actions (7). The mobile court raids against food adulteration intensified in 2005 when electronic and print media featured reports on horrendous food adulteration practices. Sixty-four percent sellers/producers in a study in Bangladesh reported using chemicals in their products, although 74% were aware that mixing chemicals with food was harmful to health. They used harmful chemicals to make the products more lucrative, increase shelf-life, substitute for unavailable natural raw materials, and reduce price of the goods (8). Recently, a growing concern has been the use of prohibited food colours, such as textile dyes in many foods to increase acceptability of food (9). Nearly half of the samples of sweetmeats and confectionary items contained non-permitted food colours in Pakistan (10). In India, consumption of non-permitted textile colours or abuse of colours were attributed to reported foodborne illnesses (11).

There are several laws and regulations in our country to ensure the standard of food manufacture and sale (Box 1) (7,12). Enforcement of food laws, rules and regulations in Bangladesh is a shared responsibility of different ministries and their concerned departments. The food samples are analyzed at different government food laboratories. The Consumer Right Protection Ordinance 2008 was passed in the Parliament on 1 April 2009.

Studies conducted on food safety mostly focus on microbiological contamination. There is limited published data on the temporal trend and magnitude of food adulteration and on consumers’ knowledge, attitude, and practices regarding food adulteration in Bangladesh. These data would help formulate preventive and control measures to reduce food adulteration and ensure the safety and integrity of food the citizens buy and consume. We conducted this study to describe the magnitude of food adulteration from 1995 to 2011 in Dhaka city; determine whether raids by the mobile food court intensifying in 2005 had any impact on food adulteration; identify commonly-adulterated food items and common adulterants; and determine food adulteration-related knowledge, attitude, and practice of consumers in Dhaka city.

## MATERIALS AND METHODS

We conducted this exploratory study in Dhaka, Bangladesh. We collected both secondary and primary data during 2006-2013 to address our study objectives.

We reviewed aggregated yearly results of food samples tested at the Public Health Food Laboratory (PHFL) of Dhaka City Corporation (DCC) from January 2001 to October 2006. The results included types and number of tested food samples, test reports as adulterated or pure, and comments. The sanitary inspectors collect both open and packaged food samples from 10 zones of DCC every month based on their suspicion, seasonal factor, and public opinion. They also collect packaged food samples with BSTI certification marks. There are 107 food items in the Pure Food Rules but all the food items are not collected for testing. The public food analyst tests the food samples according to the standard set in The Bangladesh Pure Food Ordinance, 1959 and The Bangladesh Pure Food Rules, 1967. All the tests are not done as per standard due to lack of equipment. A food sample is reported as adulterated if it does not conform to the standard. The PHFL of DCC cannot detect the nature and quantity of chemicals, artificial food colours, or quantify the presence of permitted food additives due to lack of equipment. The sanitary inspectors can suspect what substances are added to it from their experience. If a food item is found pure by testing on several occasions, it is assumed that it is not adulterated, and sample of that food item is not tested for some time. We also reviewed lay press reports on food adulteration during 1995-2011.

**Box 1. Food safety laws and regulations and food standards in Bangladesh**

The Bangladesh Pure Food Ordinance, 1959 (Bangladesh Ordinance No. LXVIII of 1959)

The Bangladesh Pure Food (Amendment) Act, 2005

The Bangladesh Pure Food Rules, 1967

Bangladesh Standards and Testing Institution Ordinance, 1985 (XXXVII of 1985)

Bangladesh Standards and Testing Institution (Amendment) Act, 2003

The Food Grain Supply (Prevention of Prejudicial Activity) Ordinance, 1956

The Essential Commodity Act, 1990

The Iodine Deficiency Disorder Prevention Act, 1989

The Animals Slaughter (Restriction) and Meat Control (Amendment) Ordinance, 1983

Fish and Fish Products (Inspection and Quality Control) Ordinance, 1983

Fish and Fish Products (Inspection and Quality Control) Rules, 1997

The Radiation Protection Act, 1987

Destructive Insects and Pests Rules, 1966 (Plant Quarantine) amended up to 1989

The Pesticide Ordinance, 1971 and The Pesticide Rules, 1985

Agricultural Produce Markets Regulation Act, 1964 (revised in 1985)

Fish Protection & Conservation Act, 1950 (latest amendment in 1995)

Marine Fisheries Ordinance, 1983 and Marine Fisheries Rules, 1983

The Special Powers Act, 1974

Agricultural Products Market Act, 1950 (revised in 1985)

We collected information from the BSTI on food sample testing results during 2005-2006 but details were unavailable due to confidentiality of the reports. We reviewed issues of ‘Consumer Voice’, the official bimonthly publication of Consumers Association of Bangladesh (CAB). We also reviewed the reports on mobile food court operations collected from a metropolitan magistrate of the Chief Metropolitan Magistrate (CMM) court conducting mobile food court raids.

We identified commonly-adulterated food items and common adulterants by reviewing the food sample testing results of the PHFL of DCC and reports on food adulteration published in the Bangla newspaper ‘The Daily Ittefaq’ and the English newspaper ‘The Daily Star’ in 2005. We collected clippings of newspaper reports from the Consumer Association of Bangladesh office where records of all news on food adulteration and other consumer-related issues published in the newspapers are regularly maintained. The reports in the newspapers are based on information collected from different stakeholders.

We conducted a cross-sectional survey in eight DCC *Kutcha* bazaars (kitchen markets) among 96 adult male and female respondents during November 2006. As data on the proportion of consumers having some knowledge on food adulteration were unavailable from published literature, our estimated sample-size was 96 for the cross-sectional survey assuming that 50% of our respondents will have knowledge about food adulteration with 95% confidence level and allowing 10% error. We included respondents residing in Dhaka for at least 2 years and not involved in food trade. From a list of *kutcha* bazaars (kitchen markets) in eight zones of DCC, we selected one *kutcha* bazaar from each zone by lottery. In each *kutcha* bazaar, we randomly selected the first consumer and, thereafter, every 5th consumer according to inclusion criteria till 12 consumers were interviewed. We administered a standardized questionnaire outside one of the entrances of the markets. We collected information on sociodemographics and food adulteration-related knowledge, attitude, and practices.

We conducted informal discussions with key stakeholders engaged in public health, including a public food analyst, a sanitary inspector, a metropolitan magistrate, and a staff at one of the DCC offices to explore relevant aspects on food adulteration.

We calculated the proportion of adulteration of each food item from the results of food sample testing at DCC laboratory. We performed chi-square test for linear trend to assess the temporal trend during 2001-2005. We performed two-sample test of proportions (Z-test) to assess the proportion of food samples found adulterated during 2005 and 2006 as an indicator of impact of mobile food court operation on food adulteration situation in 2006 compared to that in 2005. Since this is an exploratory study, we report knowledge, attitude, and practices as frequencies and percentages.

### Ethics

We secured official permission from the Chief Health Officer of DCC and the Director General of BSTI for obtaining food sample testing results. We did not collect any brand name of the tested food samples for ethical concern. We obtained informed verbal consent from the respondents before conducting the interviews. The study was approved by the Ethical Review Board of James P. Grant School of Public Health, BRAC University, Bangladesh, in collaboration with icddr,b.

## RESULTS

### Magnitude of food adulteration in Dhaka city

#### During 2001-2005

The majority of the food samples collected by the sanitary inspectors from different parts of Dhaka city and tested at the PHFL of DCC were found to be adulterated (Table 1).

Of the tested food samples, 100% of samples of the popular sweetmeat *Rosogolla* was found to be adulterated while most of the other sweetmeats, including *Sandesh, Chhana*, *Kheer,* and *Malai* were also adulterated at different levels (data not shown). *Dalda/Banaspati* (hydrogenated vegetable oil) is another food item persistently found adulterated (100% in 2001, 2003, and 2004; 97% in 2002 and 2005). Twenty-eight food items were not adulterated during 2001-2005, and 12 of these items were not tested at least for one year thereafter. No milk powder, cream, *maida* (wheat flour), fruit juice/squash, pickle, coriander seed, bread, cake, biscuit, sugar, honey, lozenge or *jorda* (smokeless tobacco) was found adulterated by the PHFL in 2005. Nine food items were not tested in the following year even after these were found to be adulterated during 2001-2004. The number of food samples tested each year varied from a single sample to 232 samples of the same food item.

**Table 1.** Food adulteration detected by Dhaka City Corporation during January 2001−December 2005

Year	No. of food items tested	No. of samples tested	No. (%) of samples adulterated
2001	37	563	422 (75)
2002	29	581	453 (78)
2003	36	960	713 (74)
2004	33	674	487 72)
2005	38	1193	763 (64)

According to the PHFL officials, conforming to a test parameter does not necessarily ensure purity of a food item. The manufacturers can manipulate by adding artificial ingredients to food items so that the test results are within acceptable limits. For example, in pasteurized milk, instead of milk protein, they can add cheaper vegetable protein, like soy protein and get test result within the expected limit.

In 2005, 12 brands of juices from 8 companies were collected from different markets of Dhaka by CAB and tested at BSTI as per Bangladesh Standards where 11 (92%) of the 12 samples had BSTI certification mark, although 8 (73%) of them failed to conform to the standard. Two orange juices had no fruit juice at all, and two juices had the preservative sulphur dioxide at more than the permitted level of 10 ppm. One brand of mango juice was substandard and did not have BSTI certification mark. The labels on the package also lacked information on percentage of the ingredients (58%), production date (25%), and expiry date (17%) (13).

Only 8 (13%) out of 62 brands of salt collected from different markets across the country by BSTI and tested at the laboratory conformed to BSTI standard (Box 2) (14). During 2005-2006, a total of 135 samples of mineral water, iodized salt, juice, soybean oil, mustard oil, and *banaspati/ghee* were collected from the market and tested. Among those, 90 (67%) did not conform to the Bangladesh Standard (BDS), and BSTI took actions, such as issuing of show-cause notice, penalty, and cancellation of license.

#### During 2001-2005

There was a statistically significant decreasing trend in the proportion of food samples that tested positive for adulteration in Dhaka city during 2001-2005 (chi-square for linear trend 39.5, p<0.001) (Figure).

#### During 1995-2011

According to the National Food Safety Laboratory under the Institute of Public Health, Bangladesh, 40-54% of daily consumed food was adulterated during 1995-2011 (15).

### Mobile court operation and its effect on food adulteration situation

During May 2005 to October 2006, a total of 2,139 mobile courts conducted anti-adulteration operations, filed 16,632 cases, sentenced 782 persons to imprisonment, and realized US$ 13,971 as fine. During 2006 (January–October), 524 samples of 34 food items were tested by DCC. Among those, 270 (52%) were adulterated. As per test results from the PHFL of the DCC, the proportion of food samples adulterated in 2006 (January–October) was lower than the proportion of food samples adulterated in 2005 (January–December) (270/524, 52% versus 763/1193, 64%, p<0.001).

**Box 2. Summary of 62 brands of salt tested for iodine by BSTI in 2005 (14)**

**Table:** 

Failed to conform to BSTI standard*	54 (87%)
Iodine content within BSTI standard of 20.0 to 50.0 ppm	33 (53%)
Iodine content <20 ppm	17 (27%)
Iodine content >50 ppm	12 (19%)
Lowest iodine content	7.4 ppm
Highest iodine content	747.6 ppm

*BSTI standards for salt other than iodine content, such as moisture, water insoluble matter, chloride content, pH range, and matter soluble in water other than sodium chloride

**Figure.**
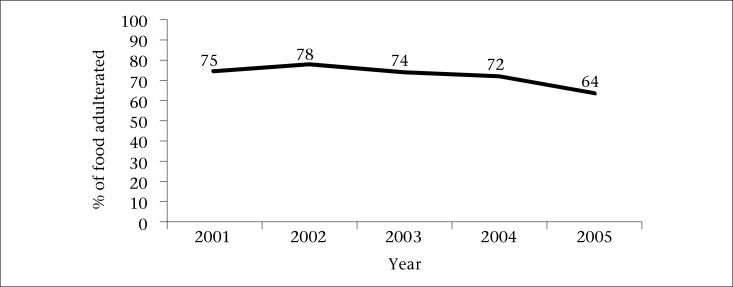
Food samples that tested positive for adulteration, detected by Dhaka City Corporation, during 2001-2005

A metropolitan magistrate conducting mobile food court activities claimed that the food adulteration situation had improved considerably, particularly maintenance of hygienic conditions and the use of expiry date on food products were noticeable. He also mentioned that the mobile court activities were sometimes hampered by non-cooperation of the field workers of BSTI and DCC. A staff at one of the DCC offices stated:

Do you think food adulteration has decreased? It has decreased only on the surface; things go on underneath. As long as corruption prevails in our country, it will not decrease. The dishonest traders get the information of mobile court raid beforehand through mobile phone.

### Adulterated food items and adulterants used

According to PHFL of DCC, 39 food items were adulterated during January 2001 to December 2005 (Box 3). Traders used various adulterants in different food items (Table 2 and Table 3) (16,17).

### Consumer awareness (knowledge, attitude, and practice)

The majority of the respondents were male (82%) (Table 4). Half of the respondents (47/94) spent more than 41% of their monthly income on food. Stale or rotten food was the most commonly-mentioned form of food adulteration by the consumers (Table 5). The majority (85%) of the respondents knew that there is a law in the country that deals with food adulteration; among them, 25 (33%) thought that the existing law and enforcement was sufficient. More than half of the respondents (53, 55%) opined that food adulteration had decreased. Only 26 (28%) respondents correctly knew how to test salt by adding it to rice and observing change in colour to purple after adding lemon juice to it (Table 6). The majority of the respondents (91, 95%) strongly agreed that food adulteration has harmful effects on health.

**Box 3. Adulterated food items in Dhaka city, 2001-2005**

**Table:** 

Milk and milk products:	Food grains, cereals, and cereal products:
Milk Dried milk powder Curd Icecream Cheese Butter Cream *Ghee* (clarified fat) Rosogolla Kalakand/Kalojam Sandesh Chhana Kheer Mawa Malai Edible oil and oil products: Butter oil Soybean oil Mustard oil Palm oil Coconut oil Dalda/Banaspati	Rice Wheat Lentil/*Dal* *Beson* (flour made of peas or chickpeas) *Ata* (course wheat flour) *Suzi* (semolina) *Lachsa semai* (kind of vermicellis) Spice: Turmeric powder Bakery products: Cake Biscuit Fruits, vegetables and miscellaneous products: Jelly Juice Sauce Pickles Ring chips Honey *Khair* (edible catechu) Salt

**Table 2.** Adulterants used in different food items of vegetable origin as reported in lay press reports, Dhaka 2006

Food category and food item	Adulterant
Edible oil	
Soybean oil	Palm oil, chemical*, colour*, burnt mobil† from rail locomotives, burnt oil from electric transformer
Mustard oil	Chemicals*
Food grain and grain products
Lentils, *mugdal, chola, mosur dal, dabli, mashkolai, buter dal* (lentil types)	Toxic colouring agents*, imported low-quality inedible lentils mixed with textile dye* and have fungal growth; less expensive *Mashkolai dal* powdered with champa colour* and sold as *mugdal*
Rice	Urea added to make it whiter
*Dhekichata chal (husked rice), ata* (course flour)	Red toxic colour* mixed with rice and *ata* to sell as husked rice, red *atta*
*Muri* (puffed rice)	Urea fertilizer to make it whiter and puffier
Wheat, corn	Animal feed packaged as human food
*Semai* (vermicellis)‡	*Dalda* made with rotten potato, cow intestine, low-quality palm oil
Vegetables and tubers	
Vegetables	Organophosphorus compounds and other pesticides
Tomato	Calcium carbide for artificial ripening
*Potol* (pointed gourd), peas	Textile dye*
Eggplant	Pesticide*
Green peas	Chemically coloured* *Dabli*
Potato	Red toxic colour*
Spices	
Mixed spices (powder)	Brick dust, saw dust, *chaler kura* (dust from outer layer of rice)
Turmeric powder	Brick dust, *buter dal, kheshari dal* (lentils), artificial powder, colour*
Chili powder	Powder with colour*
Coriander powder	C*haler kura* (dust from outer layer of rice), toxic colour*
*Zeera* (cumin) powder	Brick dust, toxic colour*, powder*
Pepper	Papaya seed
Salt	No iodine
Bakery products	
Cake‡	Textile dye, chemicals*, inedible date expired *ata/maida*, fertilizer urea, substandard inedible *dalda*, rotten egg
Biscuit‡	Ammonium bicarbonate, sodium cyclamate, fertilizer urea, toxic colouring agents*, palm oil, burnt oil, outdated inedible *ata/maida*
Bread‡	Rotten egg, outdated *ata/maida*
Fruit and fruit products	
Mango, banana, pineapple	Calcium carbide for artificial ripening
*Koromcha* (*Carissa carandas*, Christ's thorn)	Chemical colour* to sell as cherry
Orange and lychee juice	Water, flavour, textile dye*, sweet pumpkin, and colour*
Food category and food item	Adulterant
Imported juices	Substandard, date expired
Snacks	
Noodles‡	*Dhekichata chal, lal atta* (coarse wheat flour), red potato
*Chanachur*	Fried in burnt mobil†, no potato, imported powder, and colour*
*Peyaju*, *beguni*	Toxic dye*
Chocolate, sugar, and honey
Chocolate	Powder, sugar, colour*, chemical*
Sugar	Soda used instead of sugar in food
Honey	Sugar syrup
Others	
Pickle	Inedible ingredients
*Jorda* (smokeless tobacco)	Wood dust, chemical*
Mineral water and drinking-water‡	Tap-water, arsenic contaminated, contaminated with bacteria, no mineral

*Chemical nature/composition not mentioned/specified;

†Polychlorinated biphenyl (PCB) used as coolant in automobiles and transformers;

‡Prepared in unhygienic condition

Only 11 (12%) respondents considered BSTI approval when buying packaged food items while quality or freshness was commonly considered during buying open food items (Table 7). Of the respondents, 89 (93%) stated that they stopped/would stop consuming a food item if they had learnt that it was adulterated, four (4%) would continue to consume it while two (2%) would continue to consume since they had no alternative. If they suspected any food item to be adulterated while buying, 87 (91%) mentioned that they bought pure food (i.e. supposedly unadulterated) from another seller. Only 9 (9%) respondents complained it to the seller and bought pure food from another seller.

More than half of the respondents (54, 56%) mentioned using two sources of information regarding food adulteration. Television (77%), newspapers (47%), friends/neighbours (26%), co-consumers/co-buyers (10%), and radio (2%) were the sources of information. Of the respondents, 79 (82%) shared the information with others if they found any adulterated food item while 16 (17%) did not share the information.

## DISCUSSION

This exploratory study assessed the magnitude and temporal trend of food adulteration in Dhaka city as well as consumers’ knowledge, attitude, and practice relating to it. The results of the study revealed a significant decreasing trend in the proportion of laboratory-confirmed adulterated food samples during 2001-2005. However, there is no room for complacency as the extent of food adulteration remained 40-54% during 1995-2011. Nevertheless, our findings further suggest that there was substantial variation in the collected food items and the number of samples tested each year. The lack of systematic procedure for random sampling may result in potentially biased estimation of food adulteration.

Adulteration of particular food items is still rampant. *Rosogolla*, one of the most popular sweetmeats in Bengali culture, has been consistently found to be 100% adulterated. A comparative study on the quality of laboratory-made and local market *rosogolla* conducted in Mymensingh also found the laboratory-made *rosogolla* to be of better quality than ones in the market in terms of physical and chemical parameters. *Rosogolla* from the market having higher total solids and carbohydrate content with lower protein and fat level might have been due to adulteration by addition of skimmed milk *chhana*, wheat flour, and high level of sugar (18). Ninety percent of randomly-collected samples of sweetmeats from different parts of Bangladesh were also found adulterated in 2002 (6); 97% of randomly-collected 400 samples of sweetmeats from Dhaka in 2003 were adulterated, and 91% samples had less than 10% milk fat which should have been at least 10% as per Bangladesh standard laid down by BSTI and The Bangladesh Pure Food Rules, 1967 (19).

**Table 3.** Adulterants used in different food items of animal origin as reported in lay press reports, Dhaka 2006

Food category and food item	Adulterant
Egg, fish, meat, and meat products
Hen egg	White eggs of farm hens coloured red with textile dye* to sell as local hen eggs. Tortoise eggs sold as hen eggs
Fish	Inject formalin through the gills or dip fishes in water treated with chemicals, such as chlorofluoro carbon; DDT† powder to prevent rotting; add red colour* to give fresh look; sell rotten fish
Dry fish	DDT†
Mutton	Buffalo, sheep and beef meat sold as mutton
Beef	Buffalo meat sold as beef
*Halim*‡	Left over bones, intestine
Sweetmeats and dairy products
Butter	Cow's intestine, *dalda* mixed with colour*, powder*
*Ghee, dalda* (hydrogenated vegetable oil)‡	*Banaspati*, toxic chemical*, potato smash, cow's fat, intestine
Sweetened curd‡	Textile dye*
Sweetmeats‡	Textile dye named ‘thousand power colour’ and toxic chemicals*; rotten eggs; *dalda* made with cow's intestine, saccharin, soybean oil and vegetable oil instead of milk fat; paste of ground rice and sulphuric acid mixed with milk to make posset
*Jilapi* (coil-like, juicy sweet)	Fried with mobil¶
Halua	Rotten carrot and *lau* (bottle gourd), chemical*
Icecream†	Unsold foul-smelling icecream refined and re-packaged, almost no milk, palm oil for soap manufacturing, textile dye*, low-quality milk powder, sodium cyclamate
Imported milk powder	Adulterated, low-quality, date expired, without BSTI approval
Fast food and restaurant food†
Jelly, sauce	Toxic colouring agents*, chemicals*, spirit
Chicken	Dead chicken; cooked and raw meat refrigerated together
Shrimp	Sold rotten
Fish	Fried and raw fish refrigerated together

*Chemical nature/composition not mentioned/specified;

†Dichloro-diphenyl trichloroethane;

‡Prepared in unhygienic condition;

¶Polychlorinated biphenyl (PCB) used as coolant in automobiles and transformers

While no juice, fruit syrup, or fruit squash was found adulterated in DCC testing in 2005, two-thirds of the mango and orange juice samples collected by CAB and tested at BSTI were found not conforming to the BSTI standard, although the majority of the juices had BSTI certification mark. It is notable that, in the same year, the majority of the tested brands of iodized salt did not conform to BSTI standard, although half of those had the iodine content within BSTI requirements. Manufacturers do not always maintain the quality of marketed food products after receiving the certification mark from BSTI that may be intentional for more profit or due to ignorance. Since the packaged food items are produced and marketed throughout the country, quality control of marketed food products clearly warrants more stringent monitoring.

Although mobile court raids against food adulteration were not uncommon in Dhaka city, they attained momentum in 2005 when anti-adulteration drives were intensified. We found that the proportion of adulterated food samples reduced significantly in 2006 compared to that in 2005. This may be attributed to the intensified activities of the mobile food court. It was also affirmed by one of the metropolitan magistrates conducting the mobile court. Moreover, amendment of the age-old Pure Food Ordinance and media attention with reporters accompanying the magistrates and reporting revelation of horrendous practices may also have role in it. Contrary to this, one of the staff members at a DCC office claimed that this success was only superficial. The magistrate also mentioned occasional non-cooperation from the field workers of DCC and BSTI during the raids. The allegation of the alliance of dishonest traders with administrative and law-enforcing agencies has long been identified as one of the obstacles in curbing food adulteration (9,20). Nevertheless, anti-adulteration drive by the mobile court should be regularly conducted to curb food adulteration.

**Table 4.** Sociodemographic characteristics of the respondents, Dhaka, Bangladesh, 2006

Characteristics	Total sample (N=96) n (%)
Male	79 (82)
Median (range) age in years	42 (20-76)
Education	
Some schooling but did not complete secondary	17 (18)
Completed SSC	15 (15)
Completed higher secondary schooling (HSC)	21 (22)
Graduation and above	43 (45)
Occupation	
Service	50 (52)
Business	19 (20)
Household work	11 (12)
Others	16 (16)
Median (range) duration of stay in Dhaka (years)	20 (2-76)
Median (range) monthly household income (Taka)*	15,000 (3,000-150,000)
Median (range) percentage of income spent on food*	41.64 (10-83)

*Two respondents did not know

A number of food items were not found to be adulterated in the DCC food testing, although unhygienic conditions were found during manufacturing some of the food items, rendering them prone to microbiological contamination. Furthermore, anecdotes of mobile food court raids showed vermicellis being prepared in extremely unhygienic condition, keeping broom and sweaty shirts over them with flies buzzing around (21). The likely explanation for often doubtful results from DCC laboratory is the bias in sampling or technological limitations. Different colouring agents and textile dyes were extensively used in a variety of food items but detection of the nature and level of the colouring agents has not been possible due to the lack of advanced laboratory facilities. A study in India found synthetic food colour exceeding the statutory limits in the majority of food items and non-permitted colours in some of the foods sold at kiosks (22). Developing laboratory capacity for essential chemical analysis would enable to detect the nature and extent of different colouring agents and textile dyes.

Chemicals, such as calcium carbide, formalin, sodium cyclamate, DDT, and urea, were widely used. Formalin was detected in 9 out of 11 fishes tested in the Pharmaceutical Technology Department of the University of Dhaka (19). Calcium carbide, banned in 2005, is widely used for artificial ripening of fruits. Calcium carbide produces acetylene, a precursor of ethylene that has long been used for postharvest ripening of fruits as a plant hormone or growth regulator (23). Research showed that “the changes produced by ethylene are solely those that would have been brought about by nature in somewhat longer time and less uniformly.” Ethylene-treated fruits have similar qualities to naturally-ripened fruits and are harmless (24). Fruits attaining only physiological green maturity stage respond to the external ethylene (23). In the USA, both ethylene and calcium carbide are included in the National List of Allowed Synthetics (25). However, our farmers and food traders might have benefited if they were educated to harvest fruits after green maturity and judicious use of calcium carbide or ethylene thereafter (25). Polychlorinated biphenyl (PCB), commonly known as ‘mobil’, was found to be mixed with edible oil and used for frying different food items. This finding is comparable to the findings of the Department of Environment where PCB was detected in popular snacks, such as *peaju*, *jilapi*, *puri*, and potato *chops* sold in roadside shops and in packaged *chanachur* sold in stores (26). Traces of PCB were also found in blood sample of some people, although the safety level is not determined yet.

**Table 5.** Knowledge of the respondents on food adulteration, Dhaka, Bangladesh, 2006

Knowledge	Total sample (N=96) n (%)
What food adulteration constitutes†	
Stale/rotten food	23 (24)
Mixing substances harmful to health	18 (19)
Substandard food	16 (17)
Adding chemicals to food	15 (16)
Adding artificial colour to food	12 (13)
Impure food	1 (1)
Did not know	9 (9)
Food item commonly adulterated*	
Oil	43 (45)
Fish	33 (34)
Vegetables	31 (32)
Fruits	19 (20)
Spices	18 (19)
Milk	15 (16)
*Maida/Ata* (wheat flour)	10 (10)
*Muri* (puffed rice)	9 (9)
*Dal* (lentil)	8 (8)
Juices	7 (7)
Rice	6 (6)
Sugar	6 (6)
Sweetmeats	6 (6)
*Ghee*	3 (3)
Salt	3 (3)
Fast food	2 (2)
Bakery	1 (1)
Hotel food	1 (1)
Did not know	4 (4)
Common adulterants*‡	
Colour	40 (42)
Urea	22 (23)
Formalin	21 (22)
Chemical	17 (18)
Pebbles	4 (4)
Brick dust	2 (2)
Carbide	1 (1)
Sand	1 (1)
Water	1 (1)
Did not know	13 (14)

*Multiple responses;

†Two respondents did not respond;

‡One respondent did not respond; Percentages do not sum up to 100

Although food adulteration received considerable media attention, the consumers in our study lacked knowledge on what comprises food adulteration, commonly adulterated food items, and the adulterants used. A survey on awareness of consumer right found that most of the consumers knew about toxic colours and chemicals in fruits and vegetables; 85% study participants knew that there exist laws in the country that dealt with food adulteration but 67% of them opined that the existing law and enforcement was insufficient to curb the problem (27). In contrast, more than half of the respondents in this study thought that food adulteration has decreased following mobile court raids while one-third thought it has remained unchanged. The practice of considering BSTI approval was substantially low among our respondents. Consumers should be made more aware of the quality of the food they should consider while buying different food items.

### Limitations

This study has several limitations. The magnitude of food adulteration was ascertained by review of the food testing results of DCC public health food laboratory and review of CAB publications. Since the food samples are not randomly collected, there is a chance of potential bias. Due to sensitivity of the problem and, hence, inaccessibility to the details of the food testing reports, a more comprehensive scenario of adulteration could not be assessed. Although there are multiple laboratories for food analysis, results from a single laboratory were reviewed for reasons of accessibility and time constraints. The survey on consumer awareness was conducted in kitchen markets under Dhaka City Corporation. It is assumed that the people who buy food items from these markets have better income than those who do not go there. However, it is generally assumed that people of lower economic group, who struggle for their subsistence, are not concerned with the safety and quality of food they consume. Moreover, there are many small bazaars in each locality from where many consumers buy their food items. Some of the markets had more than one entrance, and it was not possible to cover all the entrances. As a result, the knowledge, attitude, and practice of the consumers interviewed may not represent that of Dhaka city dwellers and need further large-scale survey.

**Table 6.** Knowledge and attitude of the respondents on food adulteration law, current adulteration situation, and testing for salt and fish, Dhaka, Bangladesh, 2006

Characteristics	n (%)
Was there any food adulteration-related law in the country? (N=96)	
Yes	82 (85)
No	5 (5)
Did not know	9 (9)
What was in the law? (N=82)	
Provision for punishment	44 (54)
Mobile court raid	23 (28)
Punishment and mobile court raid	6 (7)
BSTI standard	3 (4)
Did not know	6 (6)
Was existing law and law enforcement sufficient to curb the problem? (N=76)	
Yes	25 (33)
No	51 (67)
Food adulteration situation following mobile court raid (N=96)	
Decreased	53 (55)
Remain unchanged	36 (38)
Increased	4 (4)
Did not know	3 (3)
Reason for decrease in food adulteration (N=53)	
Law properly enforced	43 (81)
Adequate punishment	6 (11)
Others	4 (8)
Reason for unchanged food adulteration situation (N=36)	
Reduced mobile court activity	11 (31)
Insufficient penalty	11 (31)
Political influence	9 (25)
Others	5 (14)
Know how to test salt for iodine at home (N=94)	
Knew correctly	26 (28)
Knew incorrectly	11 (12)
Did not know	57 (61)
Ever tested salt for iodine at home (N=37)	
Yes	30 (81)
No	7 (19)
Know how to identify formalin-treated fish while buying (N=96)	
Knew correctly	47 (49)
Knew incorrectly	7 (7)
Did not know	42 (44)

### Conclusions

The extent of food adulteration was high enough to warrant further action to control the situation. Collection procedure of food samples has the potential for biased estimation and, therefore, undermines the validity of the extent. A systematic procedure of random food sample collection incorporated in a surveillance for food adulteration could give an accurate picture of the situation. The extensive use of different chemicals and dyes in food calls for appropriate measures. The majority of the consumers lack proper knowledge, attitude, and practices relating to food adulteration. Publicizing the newly-passed consumer protection law, other existing food adulteration-related laws, and different aspects of food adulteration via mass media could play a crucial role in raising consumer awareness. Stringent enforcement of the forthcoming unified food law ‘Safe Food Act 2013’ by the Government would substantially decrease food adulteration in the country (28). Drives by mobile magistrate court were found to be effective and should be re-started in collaboration with the media that can publicize the results of the drive for building awareness. Epidemiologic and toxicologic studies should be undertaken for risk assessment of different synthetic food colours and chemicals used in food items to better understand short-term and long-term adverse effects on health, nutrition, and intellectual performances. Studies on epigenetics should also be performed to investigate the association of food adulteration and different types of cancer.

**Table 7.** Criteria considered by the respondents while buying packaged and open food items, Dhaka, Bangladesh, 2006

Criteria	Total sample (N=96) n (%)*
Criteria for buying packaged food	
Expiry date	32 (33)
Brand	19 (20)
Quality	17 (18)
Package	11 (12)
Price	11 (12)
BSTI approval	11 (12)
Previous experience	1 (1)
Don't know	6 (6)
Criteria for buying open food	
Quality/freshness	43 (45)
Appearance	26 (27)
Colour	15 (16)
Price	12 (13)
Cleanliness	9 (9)
Flavour	7 (7)
Don't know	4 (4)

*Multiple responses; Percentages do not sum up to 100

## ACKNOWLEDGEMENTS

This work was supported and funded by James P. Grant School of Public Health, BRAC University, Dhaka.
